# *Helicobacter pylori* CagA and IL-1*β* Promote the Epithelial-to-Mesenchymal Transition in a Nontransformed Epithelial Cell Model

**DOI:** 10.1155/2016/4969163

**Published:** 2016-07-20

**Authors:** Haruki Arévalo-Romero, Isaura Meza, Gabriela Vallejo-Flores, Ezequiel M. Fuentes-Pananá

**Affiliations:** ^1^Departamento de Biomedicina Molecular, Centro de Investigación y de Estudios Avanzados del Instituto Politécnico Nacional, Avenida Instituto Politécnico Nacional 2508, San Pedro Zacatenco, 07360 Ciudad de México, DF, Mexico; ^2^Unidad de Investigación en Virología y Cáncer, Hospital Infantil de México Federico Gómez, Dr. Márquez 162, Colonia Doctores, 06720 Ciudad de México, DF, Mexico

## Abstract

Gastric cancer is the third cause of cancer death worldwide and infection by* Helicobacter pylori* (*H. pylori*) is considered the most important risk factor, mainly by the activity of its virulence factor CagA.* H. pylori*/CagA-induced chronic inflammation triggers a series of gastric lesions of increased severity, starting with gastritis and ending with cancer. IL-1*β* has been associated with tumor development and invasiveness in different types of cancer, including gastric cancer. Currently, it is not clear if there is an association between CagA and IL-1*β* at a cellular level. In this study, we analyzed the effects of IL-1*β* and CagA on MCF-10A nontransformed cells. We found evidence that both CagA and IL-1*β* trigger the initiation of the epithelial-to-mesenchymal transition characterized by *β*-catenin nuclear translocation, increased expression of* Snail1* and* ZEB1*, downregulation of* CDH1*, and morphological changes during MCF-10A acini formation. However, only CagA induced MMP9 activity and cell invasion. Our data support that IL-1*β* and CagA target the *β*-catenin pathway, with CagA leading to acquisition of a stage related to aggressive tumors.

## 1. Introduction

Gastric cancer (GC) is the fifth most frequently diagnosed malignancy in the world and the third cause of cancer death worldwide [1]. GC is strongly associated with infection by* Helicobacter pylori* (*H. pylori*), a microaerophilic Gram-negative bacterium that persistently colonizes the gastric mucosa of at least 50% of the world's population [2]. Due to its association with GC,* H. pylori* was classified as a class 1 carcinogen by the International Agency for Research on Cancer (IARC) [3–7].

*H. pylori* expresses several virulence and colonization factors [8–11]. The pathological effects of* H. pylori* in the gastric mucosa are associated with the presence of CagA (cytotoxin associated gene A), which is encoded in the cag pathogenicity island (cagPAI), a chromosomal DNA segment of about 40 kb encoding genes for a type IV secretion system (T4SS) [6, 12–14]. Different bacteria use T4SS to release effector molecules into host cells [15, 16]. Studies in GC cell lines showed that, after the adhesion of* H. pylori* to the epithelial cell, this secretion system is used to translocate the CagA protein [17]. Once CagA is inside the cell, it is phosphorylated in EPIYA motifs by members of the Src family of kinases and by the Abelson murine leukemia viral oncogene homolog 1 (c-Abl) kinase [18–23]. Phosphorylated and unphosphorylated CagA then activate a complex network of signaling molecules directly affecting cellular process related to cellular transformation, such as proliferation, cell survival, cell polarity, and the epithelial-to-mesenchymal transition (EMT) [24–31].

Chronic inflammation is an important driver of different types of cancer [32, 33]. Particularly, GC evolves through progressive inflammatory lesions, starting with superficial gastritis, followed by atrophic gastritis, intestinal metaplasia, and dysplasia, to finally become a cancerous lesion [34, 35]. Precancerous gastric lesions are characterized by prominent infiltration of mononuclear and polymorphonuclear immune cells and the presence of inflammatory cytokines, such as tumor necrosis factor *α* (TNF*α*), interleukin- (IL-) 1*β*, and IL-8 [36]. Epidemiological data also support an association between GC and polymorphisms in the genes encoding TNF*α*, IL-1*β*, and IL-8 [37–39]. A transgenic mouse ectopically expressing IL-1*β* develops progressive lesions that mirror the multistage process occurring during human gastric carcinogenesis. Interestingly, these pathological changes are accelerated when* H. pylori* infection is introduced providing evidence that IL-1*β* and* H. pylori* can cooperate during gastric carcinogenesis [40–44].

EMT is characterized by multiple transcriptional, biochemical, and morphological changes allowing a terminally differentiated epithelial cell to acquire a mesenchymal phenotype. Tumor cells become migratory after EMT, with increased capacity for degrading extracellular matrix (ECM) components and resistance to anoikis, a type of apoptosis of detached cells [45]. It is believed that progression from noninvasive to invasive tumors relies on activation of the EMT program characterized by activation of transcription factors Snail, Twist, Slug, and ZEB and loss of cell-to-cell junctional complexes induced by nuclear translocation of *β*-catenin, degradation of E-cadherin, and downregulation of the* CDH1* promoter. All together these processes associate with highly aggressive tumors [46].

We recently found that CagA induces anoikis resistance through activation of the AKT signaling pathway [47]. AKT is also an important mediator of EMT through inactivation of glycogen synthase kinase 3*β* (GSK3*β*), a critical negative regulator of *β*-catenin. Once GSK3*β* is inactive, *β*-catenin moves to the nucleus where it helps to turn on the EMT transcriptional program [48]. Recent studies have also supported a link between CagA, AKT, and EMT [49, 50]. In this study, using a nontransformed epithelial cell model we found that both CagA and IL-1*β* induced translocation of *β*-catenin to the nucleus and the onset of EMT, but only CagA led to enhanced MMP9 activity and cell invasion.

## 2. Material and Methods

### 2.1. *H. pylori* Strains and Culture

Two CagA positive* H. pylori* strains were used in this study: strain 11637 with a Western type CagA (EPIYA ABCCC) that was obtained from the American Type Culture Collection (ATCC, Manassas, VA, USA, number 43504) and strain NY02-149 with an East-Asian-type CagA (EPIYA ABD) that was kindly donated by Dr. Guillermo Perez-Perez from New York University. Two additional* H. pylori* CagA negative strains were used as controls: strain 365A3, which has a partial cagPAI lacking the* cagA* gene, and strain 254 that contains a nonfunctional cagPAI. All* H. pylori* strains were grown on blood agar (BD Biosciences, San Jose, CA, USA, number 211037) for 48 h at 5% CO_2_ and 37°C.

### 2.2. MCF-10A Culture, Infection, and IL-1*β* Stimulation

MCF-10A cells are human mammary epithelial cells that were obtained from the American Type Culture Collection (ATCC CRL-10317, Manassas, VA, USA). MCF-10A three-dimensional (3D) or monolayer (2D) cultures were performed as previously reported by us and Debnath et al. [47, 51]. For infection assays, MCF-10A cells were seeded at 3000 cells/cm^2^ and cultured for 48 hrs to reach 70% subconfluency and then switched to DMEM-F12 without fetal bovine serum (FBS). Afterwards, cells were infected with an* H. pylori* multiplicity of infection (MOI) of 100 and/or stimulated with 20 ng/mL of human recombinant IL-1*β* (Peprotech, Rocky Hill, NJ, USA) for 48 hrs. As a control for all experiments, cells were similarly handled but were not infected nor treated with IL-1*β* (mock infected/treated control cells). For 3D culture infection-stimulation assays, single cells suspensions were treated as mentioned above and then seeded in Matrigel (BD Biosciences, San Jose, CA, USA, number 354230). Cells were grown for 14 days changing medium every other day. In infected and/or IL-1*β* treated cells, media of days 2, 4, and 6 also contained bacteria and IL-1*β* to maintain the stimulus while the acini were growing and shaping.

### 2.3. Immunofluorescence

Cells grown under 3D conditions were fixed with paraformaldehyde (PFA Electron Microscopy Sciences Cat. 15713) at 3.7% for 20 minutes at room temperature, washed, and permeabilized with PBS-0.2% Triton X-100 for 20 minutes and then washed again and treated with PBS-0.02% Triton X-100 plus 10% goat-serum and 1% BSA (blocking buffer). Cells were then incubated overnight with anti-GM130 antibody (Genetex number GTX61445; 1 : 50 dilution in blocking buffer), washed, and incubated with anti-rabbit IgG labeled with Alexa-488 (Invitrogen, Carlsbad, CA, USA, number A11008, 1 : 100 dilution) for one hour. Cells were then stained with DAPI (Invitrogen, Carlsbad, CA, USA, number D1306). Finally, the preparations were washed and mounted in the 3D culture chambers adding 12 *μ*L of VECTASHIELD (Vector Laboratories, Burlingame, CA, number H-1000) and observed in a confocal microscope Leica SP2 (Leica Microsystems, Wetzlar, Germany). Confocal images were analyzed with the Leica LAS AF-Lite 2.6.0 software.

For 2D assays, cells were grown on sterile glass coverslips in DMEM-F12 for 48 hours. Cells were then serum starved and IL-1*β*-stimulated or infected for 48 hours. Then, cells were subjected to the same staining process as described for the 3D cultures by using antibodies against *β*-catenin (Invitrogen, Carlsbad, CA, USA, number 138400), E-cadherin (BD Biosciences, San Jose, CA, number 610182), and ZO-1 (Genetex, Irvine, CA, number GTX108613). Finally, cell preparations were mounted on slides and observed with the inverted epifluorescence microscope Olympus IX50. Images were recorded with the DP72 digital camera and analyzed with Image-Pro Plus software (V7.0) averaged cybernetics (Silver Spring, MD, USA).

### 2.4. RNA Extraction and RT-PCR

Total RNA was obtained from IL-1*β* stimulated and* H. pylori *infected cells lysed with 1 mL of TRIzol (Invitrogen, Carlsbad, CA, USA, number 15596018). Complementary DNA synthesis was performed using 2.5 *μ*g of total RNA in a reaction mixture with SuperScript Kit VILO Master Mix (Invitrogen, Carlsbad, CA, USA, number 11755-050).* ZEB1* gene was amplified by using the oligonucleotide pairs, sense: GGG AAT GCT AAG AAC TGC TGG and antisense: GGT GTA ACT GCA CAG GGA GC. For* Snail1* gene the oligonucleotides were as follows: sense: TCG GAA GCC TAA CTA CAG CGA and antisense: AGA TGA GCA TTG GCA GCG AG; for* CDH1* sense: CCC ACC ACG TAC AAG GGT C and antisense: CTG GGG TAT TGG GGG GCA TC; for* RPLP0* sense: ATG GGG AAG CTG AAG GTC GG and antisense: GTG GCA GTG ATG GCA TGG ACT; for* GAPDH* sense: ATG GGG AAG GTG AAG GTC GG and antisense: GTG GCA GTG ATG GCA TGG ACT. The 20 *μ*L PCR mixture contained 200 *μ*M of dNTPs mix, 2.0 mM of MgCl_2_, 200 nM of each primer, Taq Polymerase buffer, and 1.0 U of recombinant Taq Polymerase (Invitrogen, Carlsbad, CA, USA, number 11615-010). The reaction was performed with an initial denaturation step at 94°C for 2 min, followed by 30 cycles of 94°C for 1 min, 60°C for 1 min, and 72°C for 1 min, and a final extension of 72°C for 5 min.

### 2.5. Zymography

MCF-10A cells were infected with* H. pylori* or treated with IL-1*β* for 48 h and culture supernatants were recovered and concentrated using 30 K cutoff Amicon Centricon filters (Millipore, Billerica, MA, USA, number UFC503024). Protease activity was revealed in 8% SDS-PAGE gels copolymerized with 1 mg/mL gelatin and activation buffer (50 mM Tris-HCl, pH 7.4, 4.5 mM CaCl_2_). Gels were stained with Coomassie Blue and densitometric analyses were performed with the ImageJ software.

### 2.6. Invasion Assays

1 × 10^3^ IL-1*β*-stimulated or* H. pylori* infected MCF-10A cells were seeded in the inner chamber of Transwell units (Corning, NY, USA, number 3422) with Matrigel-coated polycarbonate filters as a substrate for degradation and filled up with DMEM-F12 free of serum. The outer chamber of the Transwell unit was loaded with DMEM-F12 supplemented with 10% of FBS as the chemoattractant. Cells were allowed to migrate for 36 h at 37°C. The porous membranes containing migrating cells were cut out from the inner chambers and fixed with 3.4% PFA for 30 min. Fixed cells were permeabilized with PBS-0.2% Triton X-100 and stained with DAPI.

### 2.7. SDS-PAGE and Western Blot

30 *μ*g of protein extracts was separated in 10% sodium dodecyl sulfate polyacrylamide gels (SDS-PAGE) and then transferred onto nitrocellulose membranes. Membranes were blotted with antibodies against E-cadherin, *β*-catenin, and *β*-actin as loading control (kindly provided by Dr. J. M. Hernandez; CINVESTAV-IPN, Mexico). HRP-conjugated secondary antibody was from Invitrogen (Carlsbad, CA, USA, number G21040). Positive bands were revealed by enhanced chemiluminescence.

### 2.8. Statistical Analysis

Statistical analyses were performed by one-way ANOVA test. Data are presented as mean ± standard deviation. *p* values ≤ 0.05 were considered significant.

## 3. Results

**Figure 1:**
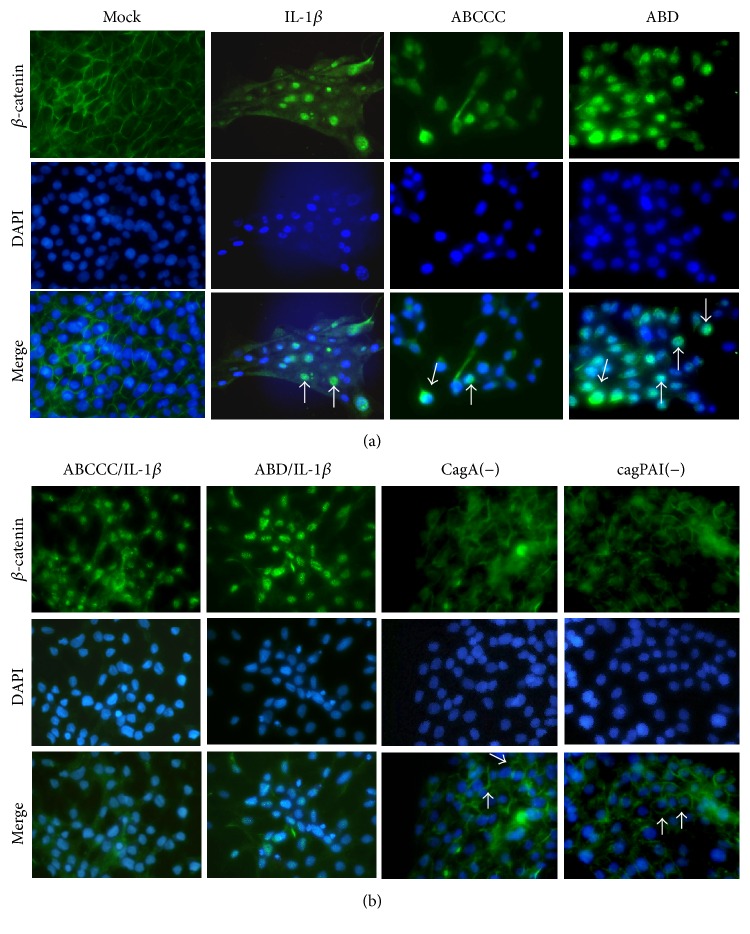
*H. pylori* CagA and IL-1*β* induce *β*-catenin nuclear translocation. (a) MCF-10A cells were infected with CagA positive strains (ABCCC or ABD) or stimulated with IL-1*β*. (b) MCF-10A cells were infected with CagA positive strains and stimulated with IL-1*β* or single infected with CagA negative variants CagA(−) and cagPAI(−). Immunofluorescence images show *β*-catenin (green) and nuclei (DAPI, blue). Arrows indicate nuclear staining (a) and membrane staining (b) of *β*-catenin. Figures are representative of three independent experiments performed in duplicate or triplicate.

We addressed whether CagA alone and/or cooperating with the inflammatory cytokine IL-1*β* promotes *β*-catenin translocation, one of the first steps of EMT. In mock control cells *β*-catenin was localized to cellular membrane. In contrast, after IL-1*β* treatment or infection with* cagA* positive strains we observed that *β*-catenin was translocated to the nucleus (Figure 1(a)). We did not find evidence that the combination of the two stimuli was able to induce the translocation of *β*-catenin into the nuclei of cells with an additive or synergistic effect (Figure 1(b)). When cells were infected with* H. pylori* strains lacking* cagA* or with a defective cagPAI, the membrane localization of *β*-catenin was not altered (Figure 1(b)).

**Figure 2:**
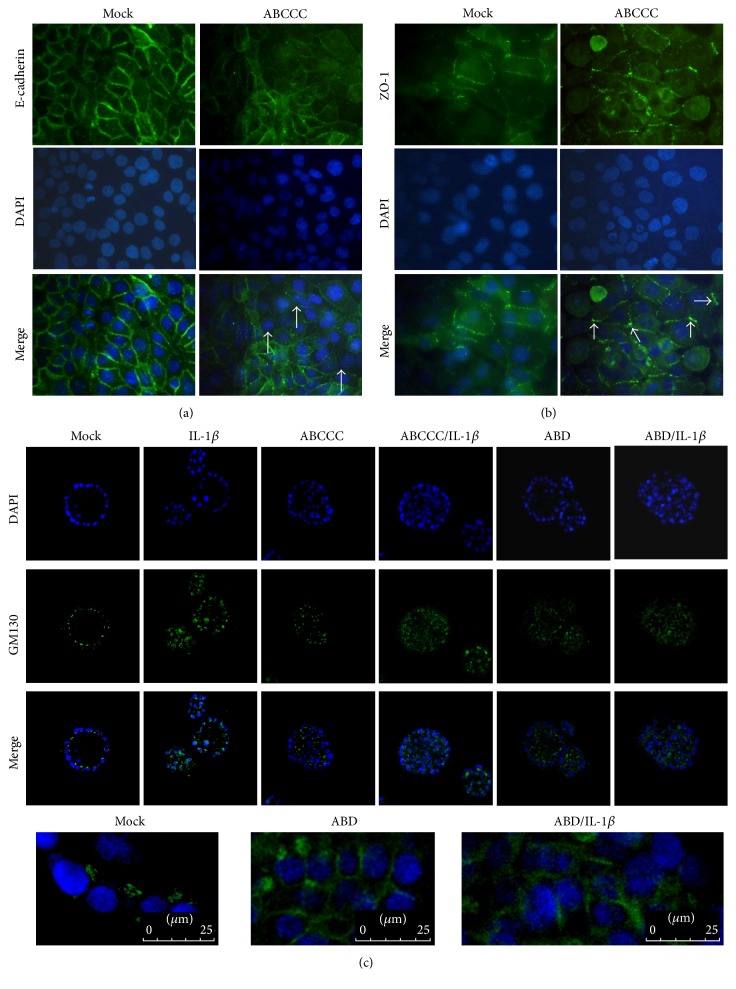
Localization of ZO-1 and GM130 is altered by* H. pylori* CagA. (a-b) Cells were infected with the CagA positive strain EPIYA ABCCC and were stained with anti-ZO-1 or anti-E-cadherin antibodies (green) and DAPI (blue). Arrows in panel (a) indicate the loss of signal of E-cadherin and in panel (b) show delocalization of ZO-1. (c) MCF-10A cells were infected with* H. pylori* positive strains ABCCC and ABD, stimulated with IL-1*β*, or both infected and stimulated during acini morphogenesis. The acini structures were stained with anti-GM130 antibody (green) and DAPI (blue) and confocal microscopy sections were made at 50% depth of the acini. The bottom panels show an enhanced fragment of the acini to see in more detail the GM130 distribution. Figures are representative of three independent experiments performed in duplicate or triplicate.

We analyzed the effect of CagA on other membrane proteins located at adherent junctions. We found that E-cadherin was distributed on the cell membrane homogeneously in uninfected cells, while in cells infected with the* cagA* positive strains there were some areas in which the fluorescent signal decreased (arrows) ((Figure 2(a)). CagA was also able to redistribute ZO-1 (arrows) (Figure 2(b)). We then evaluated the effect of CagA and IL-1*β* on MCF-10A 3D acini morphogenesis. In mock cells, GM130 localized towards the center of the acini in the apical face. However, when cells were stimulated with IL-1*β* or infected with* H. pylori cagA* positive strains a redistribution of GM130 protein was observed surrounding acini central cells (Figure 2(c) top panels and bottom enhanced images). These results show that CagA and IL-1*β* interfere with GM130 localization during acini morphogenesis.

**Figure 3:**
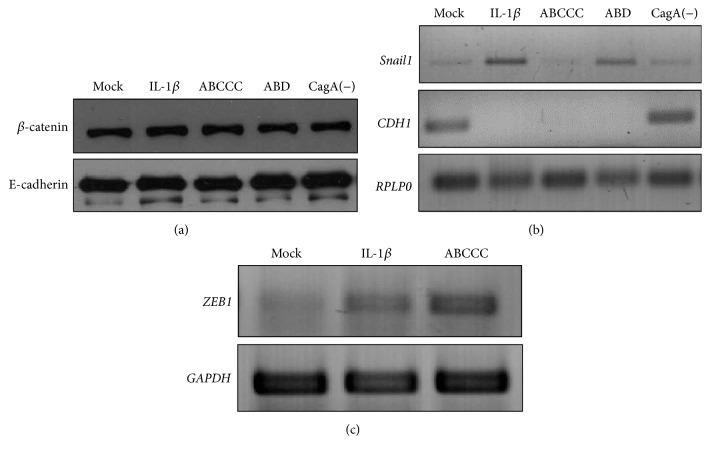
Nuclear translocation of *β*-catenin correlates with increased expression of* ZEB1* and* Snail1*. (a) Immunoblotting of whole cell lysates of infected or IL-1*β*-stimulated cells with anti-*β*-catenin and anti-E-cadherin antibodies. The relative expression of (b)* Snail1* and* CDH1* and (c)* ZEB1* genes was determined by semiquantitative RT-PCR. Expression of housekeeping genes* RPLP0* and* GAPDH* was used as internal control. Figures are representative of three independent experiments performed in duplicate or triplicate. Figures are representative of three independent experiments performed in duplicate or triplicate.

When *β*-catenin and E-cadherin were analyzed by Western blot, we observed that both proteins remain stable after both CagA and IL-1*β* stimuli at early experimental time-points (Figure 3(a)). On the other hand, the expression levels of transcription factors* ZEB1* and* Snail1*, two of the master regulators of EMT initiation, and of* CDH1*, the gene that encodes for E-cadherin, were significantly changed after both stimuli. We found an increased expression of* Snail1* and* ZEB1* after stimulation with IL-1*β* or infection (Figures 3(b) and 3(c)), but* Snail1* expression was significantly larger for the* H. pylori* with the East Asian EPIYA (ABD) than with the Western ABCCC strain, while* CDH1* expression was severely diminished by IL-1*β* treatment or infection with any of the* cagA* positive* H. pylori* strains (Figure 3(b)).

**Figure 4:**
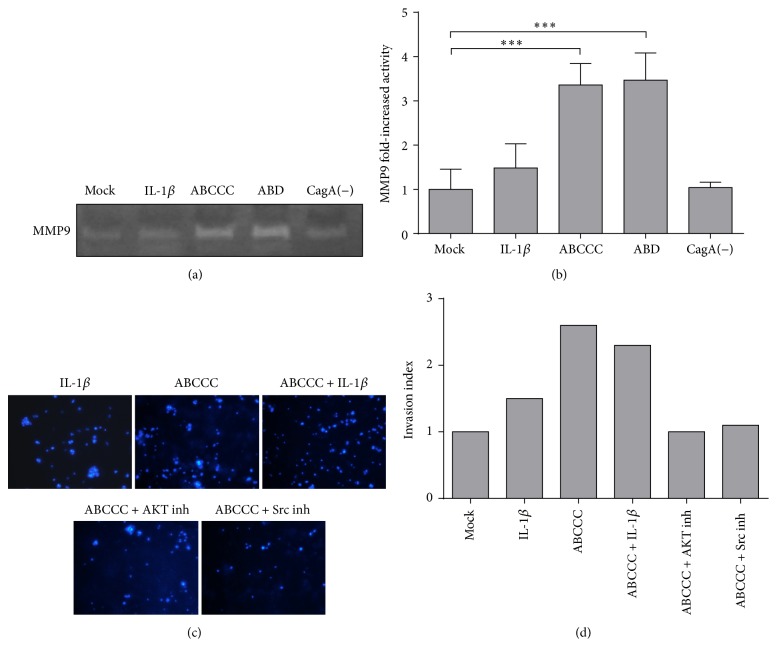
CagA but not IL-1*β* promotes MMP9 activity and invasion. (a) Representative gelatin zymogram showing metalloproteinase 9 (MMP-9) hydrolytic activity in culture media recovered from MCF-10A cells stimulated with IL-1*β* or infected with* cagA* positive and negative* H. pylori* strains. (b) Densitometric analysis of three independent experiments. (c-d) Invasion assays of cells stimulated with IL-1*β* or infected. AKT and Src kinase inhibitors (inh) were used to inhibit the epithelial-to-mesenchymal transition (EMT) and CagA activity, respectively. (c) shows a representative insert from which the invading cells were counted and (d) shows a graphical representation of the quantification of all assays. The bars are the mean fold changes normalized to mock cells. Experiments for (a) and (b) were done three times in duplicate and for (c) and (d) once in triplicate. Statistical analyses were performed by one-way ANOVA (^*∗∗∗*^ *p* ≤ 0.001) and mock cells were used as the baseline value.

We then measured the activity of metalloproteases secreted in the supernatants of MCF-10A cells. Densitometric analysis of hydrolyzed bands in gelatin zymograms showed enzymatic activity with a molecular weight correlating with that of MMP9 (Figure 4(a)). This activity had a major peak when cells were subjected to* H. pylori* infection compared to that after IL-1*β* stimulus (Figure 4(b)). An invasion assay showed that CagA has the ability to confer migratory properties on* H. pylori* infected MCF-10A cells (Figures 4(c) and 4(d)). Interestingly, similar to the metalloproteinase assay, IL-1*β* was not as efficient to induce cell invasion. Finally, we found that the stimulation of invasion is dependent on AKT and Src kinase activities (Figures 4(c) and 4(d)). Src is the main kinase that phosphorylates CagA and we have previously shown that CagA-induced activation of AKT relies on Src activity [47]. Overall these results agree with a CagA and IL-1*β*-induced onset of EMT, with CagA promoting more aggressive cancer features.

## 4. Discussion

The nontransformed cell line MCF-10A recapitulates several traits of the architecture of glandular epithelium providing a system in which to ask questions about the mechanisms of cell growth, proliferation, growth factors independence, and cell polarity. This model has been used to study mechanisms of transformation triggered by viral and cellular oncogenes, including some that are not related to the development of breast carcinomas [51–54].

ZO-1, E-cadherin, and *β*-catenin are part of the epithelial apical junctional complexes that regulate cell polarity, proliferation, and differentiation [55–57].* H. pylori* infection induces loss of polarity and relocation of ZO-1 protein in MDCK cells, which causes impairment of the epithelial barrier integrity [26, 30, 58]. E-cadherin is frequently lost in EMT-induced metastatic cancer cells [59]. We observed that although protein levels of E-cadherin remained unchanged, there was downregulation of the* CDH1* transcript after both IL-1*β* and CagA stimulus. E-cadherin degradation may be activated at later times than the ones analyzed in this study or EMT may happen without affecting levels of E-cadherin as it has been shown in other studies [60, 61].

A link between CagA and EMT has been shown in previous studies. In AGS and MKN74 cells* H. pylori* induced expression of mesenchymal markers* ZEB1*,* vimentin*,* Snail1*,* Snail3*, and* MMP9* with concomitant decreasing of the epithelial marker* keratin-7* [62]. EMT also generated cells with characteristics of cancer stem cells (CSC) and expression of CD44 [63]. However, AGS and MKN74 cells are transformed; hence the signaling pathways and biological processes associated with cancer are already altered before the expression of CagA [62, 63]. In the past two years, several primary human gastric organoid models have emerged to assess* H. pylori* infection or CagA activity. In one study,* H. pylori* was found to induce secretion of TNF*α* and IL-1*β* inflammatory cytokines, as well as several chemokines, through activation of NF*κ*B [64]. In other studies,* H. pylori* altered the cell polarity through relocalization of claudin-7 by activation of *β*-catenin and Snail [65].

It is thought that resistance to anoikis is responsible for the survival of invading tumor cells upon undergoing EMT and detaching from the basal membrane [66]. We previously showed that CagA induced anoikis resistance via AKT phosphorylation and inactivation of the proapoptotic proteins BIM and BAD [47]. AKT is also an important inducer of *β*-catenin nuclear translocation through inactivation of GSK3*β*; nuclear *β*-catenin then initiates the EMT transcriptional program [67]. Other studies have shown CagA activity leading to *β*-catenin nuclear accumulation [31]. Our results show that translocation of *β*-catenin correlates with an increased expression of the* Snail1* and* ZEB1* EMT genes, which are involved in deregulation of adherens junctions by transcriptional repression of the* CDH1* promoter [68]. Furthermore, we also observed increased cell invasion and MMP9 protease activity promoted by CagA. Metalloproteinases degrade ECM components facilitating cell invasiveness. Interestingly, we only observed a CagA-mediated increased MMP9 activity and cell invasiveness, in spite of IL-1*β* efficiently promoting *β*-catenin translocation, transcriptional upregulation of* ZEB1* and* Snail1*, and downregulation of* CDH1*. This may be due to a more chronic requirement on IL-1*β* to achieve a similar activity. A recent report showed that stable EMT-related changes were only induced after >3 weeks of IL-1*β* treatment [69], while in our study treatment went only for 2 to 6 days. Also relevant is the lack of cooperation between CagA and IL-1*β*. This could be because infection by* H. pylori* may directly induce an inflammatory response in which IL-1*β* is present, thus already saturating the need for IL-1*β* activity.

## 5. Conclusions

Our findings support that CagA oncoprotein from* H. pylori* and the inflammatory stimulus of IL-1*β* guide the onset of EMT in nontransformed cells using a model of acini morphogenesis. CagA but not IL-1*β* was found to induce cell invasion and formation of an aggressive phenotype related to cancers.
